# A Versatile Strategy for the Semisynthetic Production of Ser65 Phosphorylated Ubiquitin and Its Biochemical and Structural Characterisation

**DOI:** 10.1002/cbic.201500185

**Published:** 2015-06-18

**Authors:** Cong Han, Kuan-Chuan Pao, Agne Kazlauskaite, Miratul M K Muqit, Satpal Virdee

**Affiliations:** MRC Protein Phosphorylation and Ubiquitylation Unit, College of Life Sciences, University of Dundee Dow Street, Dundee DD1 5EH (UK)

**Keywords:** enzyme catalysis, ligases, phosphorylation, synthetic methods, ubiquitin

## Abstract

Ubiquitin phosphorylation is emerging as an important regulatory layer in the ubiquitin system. This is exemplified by the phosphorylation of ubiquitin on Ser65 by the Parkinson's disease-associated kinase PINK1, which mediates the activation of the E3 ligase Parkin. Additional phosphorylation sites on ubiquitin might also have important cellular roles. Here we report a versatile strategy for preparing phosphorylated ubiquitin. We biochemically and structurally characterise semisynthetic phospho-Ser65-ubiquitin. Unexpectedly, we observed disulfide bond formation between ubiquitin molecules, and hence a novel crystal form. The method outlined provides a direct approach to study the combinatorial effects of phosphorylation on ubiquitin function. Our analysis also suggests that disulfide engineering of ubiquitin could be a useful strategy for obtaining alternative crystal forms of ubiquitin species thereby facilitating structural validation.

Post-translational modification of proteins with the small protein modifier ubiquitin (Ub) regulates many aspects of eukaryotic biology.^1^ Crosstalk with other post-translational modifications potentially serves as an additional regulatory layer in the ubiquitin system. Over the last few years, large-scale phosphoproteomic screens have identified a number of phosphorylation sites on human ubiquitin itself, at residues Thr7,^2^ Thr12,2b, 3 Thr14,3b, 4 Ser57,2a, 3, 4b, 5 Tyr59,^6^ Ser653b, 7 and Thr66.3b However, until recently their biological significance and the identity of the upstream kinases mediating this phosphorylation were unknown. Last year three groups reported that in response to mitochondrial depolarisation, PTEN-induced kinase 1 (PINK1) phosphorylates ubiquitin on Ser65.^8^ Phospho-Ser65-ubiquitin (Ub-pSer65) was demonstrated to function as a signalling molecule that was sufficient to activate the RING-in-between-RING (RBR) E3 ligase, Parkin, both in vitro and in cells, thereby leading to autophagic clearance of damaged mitochondria (“mitophagy”).^8^ Mutations in PINK1 and Parkin are causative of autosomal-recessive early onset Parkinson's disease (PD),^9^ and the elaboration of their roles in a mitochondrial quality control pathway involving Ub-pSer65 represents a significant breakthrough in the understanding of PD mechanisms. There is now significant interest in further dissecting the role of Ub-pSer65 in PD and understanding the roles of other phosphorylation sites of ubiquitin. Two acetylation sites on Ub have also been identified and acetylation at Lys6 stabilises monoubiquitylated histone H2B in cells.4a

Although Ub can be enzymatically phosphorylated in preparative quantities with recombinant PINK1,^10^ this approach is restricted to Ser65 and is not compatible with mutational studies that seek to target sites in ubiquitin that might be required for kinase recognition. Furthermore, techniques that would facilitate the production of site-specifically phosphorylated ubiquitin to allow their study in isolation and their combinatorial effects would be extremely valuable, particularly in discerning the functional significance of the other reported phosphorylation sites of ubiquitin.

We therefore sought to develop a readily adaptable platform for the enzyme-independent production of phosphorylated ubiquitin species on a multi-milligram scale. To achieve this we explored expressed protein ligation between two peptide building blocks.^11^ Although similar strategies have been developed for preparing synthetically modified ubiquitin,^12^ these procedures require extensive or specialist synthetic peptide synthesis. Furthermore, these approaches are not readily applicable to Ub tagged with fluorescent or self-labelling proteins, which are valuable experimental tools. Genetic incorporation of phosphoserine has been reported, but this does not produce sufficiently large amounts of material for biophysical or structural studies.^13^

We explored recombinant production of the N-terminal peptide building block combined with a synthetic C-terminal building block that could be readily synthesised by a commercial service. These two peptide precursors could then be ligated with a peptide bond by native chemical ligation.^14^ We chose Phe45–Ala46 as a ligation site. Thus, four of the seven phosphosites of Ub are in the synthetic portion so, in principal, could be incorporated in a combinatorial manner. This position has also been successfully used for the assembly of modified ubiquitin from entirely synthetic building blocks.12e Following ligation, Cys46 can be conveniently desulfurised to form the native Ala46 residue.12e, 15 Recombinant production of the N-terminal fragment as the requisite thioester would provide a readily adaptable route amenable to conventional site-directed mutagenic protocols and genetic code expansion technologies that direct the efficient incorporation of additional identified Ub post-translational modifications, such as chain formation and acetylation.4a, 16

To generate Ub-1-45 bearing C-terminal thioester functionality (Ub-1-45-SR; R: CH_2_CH_2_SO_3_H) we expressed ubiquitin residues 1–45 in *Escherichia coli* as an in-frame fusion with the *Mxe* GyrA intein, as described for full-length ubiquitin (Scheme 1).^17^ Ub-1-45-SR was then liberated by thiolysis of the purified fusion protein with 2-mercaptoethane sulfonic acid (MESNA). Surprisingly, the truncated Ub thioester was highly soluble, thus suggesting that the junction between residues 45 and 46 could be a novel site for technologies using complementation of split ubiquitin.^18^ Further purification and lyophilisation yielded ∼10 mg of polypeptide per litre of culture medium (Figure S1 in the Supporting Information). The relatively short peptides corresponding to residues C46–76 of Ub, without and with phosphorylated Ser65 (UbC46-76 and UbC46-76-pSer65), were commercially sourced on 5 and 25 mg scales, respectively (Figures S2 and S3). This strategy provided a scalable platform for the production of milligram quantities of ubiquitin. This, in principle, could be used to prepare protein with any combination of post-translational modification or unnatural functionality within the C-terminal 30 residues. Additionally, the N-terminal 45 residues could be readily modified by genetic approaches.

**Scheme 1:**
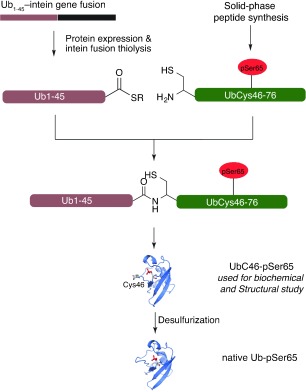
Strategy for expressed protein ligation (EPL) of site-specifically phosphorylated ubiquitin. The N-terminal fragment of Ub (residues 1–45) was obtained as a C-terminal thioester by thiolysis of a recombinant Ub_1–45_–intein fusion. The C-terminal peptide (residues 46–76 including phosphoserine at position 65) was synthesised commercially by solid-phase peptide synthesis. The native Ala46 residue was mutated to Cys to facilitate EPL; subsequent Cys desulfurisation furnishes the native protein.

In parallel, Ub-1-45-SR was ligated to UbC46-76 and UbC46-76-Ser65 in denaturing phosphate buffer by using mercaptophenylacetic acid (MPAA) as catalyst.^19^ Ligation went to near completion after 2 h incubation at 25 °C (as determined by LC-MS) to yield ubiquitin with an Ala46Cys mutation (UbC46), or Ala46Cys and phosphoserine at position 65 (UbC46-pSer65; Figure 1 A). The products were purified by reversed phase HPLC and characterised by LC-MS (Figure 1 B–D). Lyophilised UbC46 and UbC46-pSer65 were obtained in 44 % yield. The polypeptides were then dissolved in denaturing buffer and folded by dialysis against non-denaturing buffer (yield ∼95 %). In-gel tryptic digestion and LC-MS/MS analysis confirmed phosphoserine at Ser65 (Figure S4). The yield of phosphoubiquitin obtained by enzymatic phosphorylation^10^ was not reported; however, a chromatographic step was required to remove kinase and unphosphorylated material. As our ligation went to near completion and a single chromatographic step was required, the yield from our approach most likely compares favourably.

**Figure 1:**
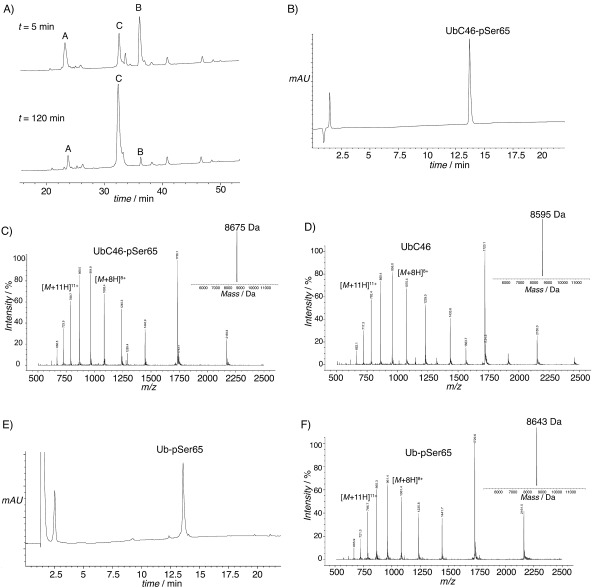
Expressed protein ligation and characterisation of phospho and non-phospho forms of Ub. A) Representative analytical HPLC of the ligation between Ub-1-45-SR (peak B) and UbC46-76 (peak A). The product, UbC46 (ubiquitin containing the Ala46Cys mutation), is observed after 5 min (peak C), and after 2 h the reaction has gone to near completion. B) HPLC analysis of UbC-pSer65 ligation product after purification by semi-preparative HPLC. C) ESI-MS spectrum of purified UbC46-pSer65; inset: deconvoluted spectrum (calcd: 8676.9 Da; found 8675 Da). D) ESI-MS spectrum for purified UbC46; inset, deconvoluted spectrum (calcd: 8596.9 Da; found 8595 Da). E) HPLC analysis of native Ub-pSer65 ligation product after free radical desulfurisation of UbC46-pSer65 for 3 h. F) ESI-MS spectrum of native Ub-pSer65 generated by desulfurisation. A single product (loss of 32 Da relative to UbC46-pSer65, 8675 Da) indicates quantitative conversion and preservation of the phosphate moiety (calcd: 8644.8 Da; found: 8643 Da).

We also determined whether conversion of Cys46 to Ala46 was possible in the presence of the phosphoserine residue, in order to obtain native phosphorylated Ub (Ub-pSer65). A sample of lyophilised UbC46-pSer65 was subjected to free-radical desulfurisation.^15^ After 3 h, quantitative conversion to Ub-pSer65 was observed by LC-MS (Figure 1 E and F). The product was then folded, and residual reaction additive was removed with a desalting column.

We next tested the cysteine mutant UbC46C and UbC46-pSer65 in biochemical assays for their ability to activate the E3 ligase Parkin.^8^ We employed a Parkin E3 ligase activity assay that monitors ubiquitylation of the substrate Miro1 as well as the formation of free polyubiquitin chains; this assay has previously been used to demonstrate activation of full- length Parkin E3 ligase activity upon addition of enzyme-derived Ub-pSer65.8a As expected, UbC46 did not lead to appreciable activation of Parkin; however, UbC46-pSer65 induced marked Parkin activation, as determined by polyubiquitin chain formation, ubiquitylation of Miro1 and Parkin autoubiquitylation (Figure 2 A). We also assessed UbC46 and UbC46-pSer65 for their ability to stimulate Parkin-mediated discharge of ubiquitin from a ubiquitin-charged E2 (UbcH7∼Ub) as previously observed for enzymatically derived Ub-pSer65.8a This assay decouples the loading of E3 with ubiquitin from transfer of ubiquitin from E3 to substrate. As expected, UbC46-pSer65 promoted Parkin-mediated ubiquitin discharge from UbcH7∼Ub, whereas UbC46 did not (Figure 2 B). In both the ubiquitylation assay and the UbcH7∼Ub discharge assay, Parkin activation was not as high as observed with wild-type Ub that had been phosphorylated by PINK1.8a The minimal activation of Parkin by UbC46 is most likely attributable to the Ala46Cys mutation (which can be converted to the native Ala).12e

**Figure 2:**
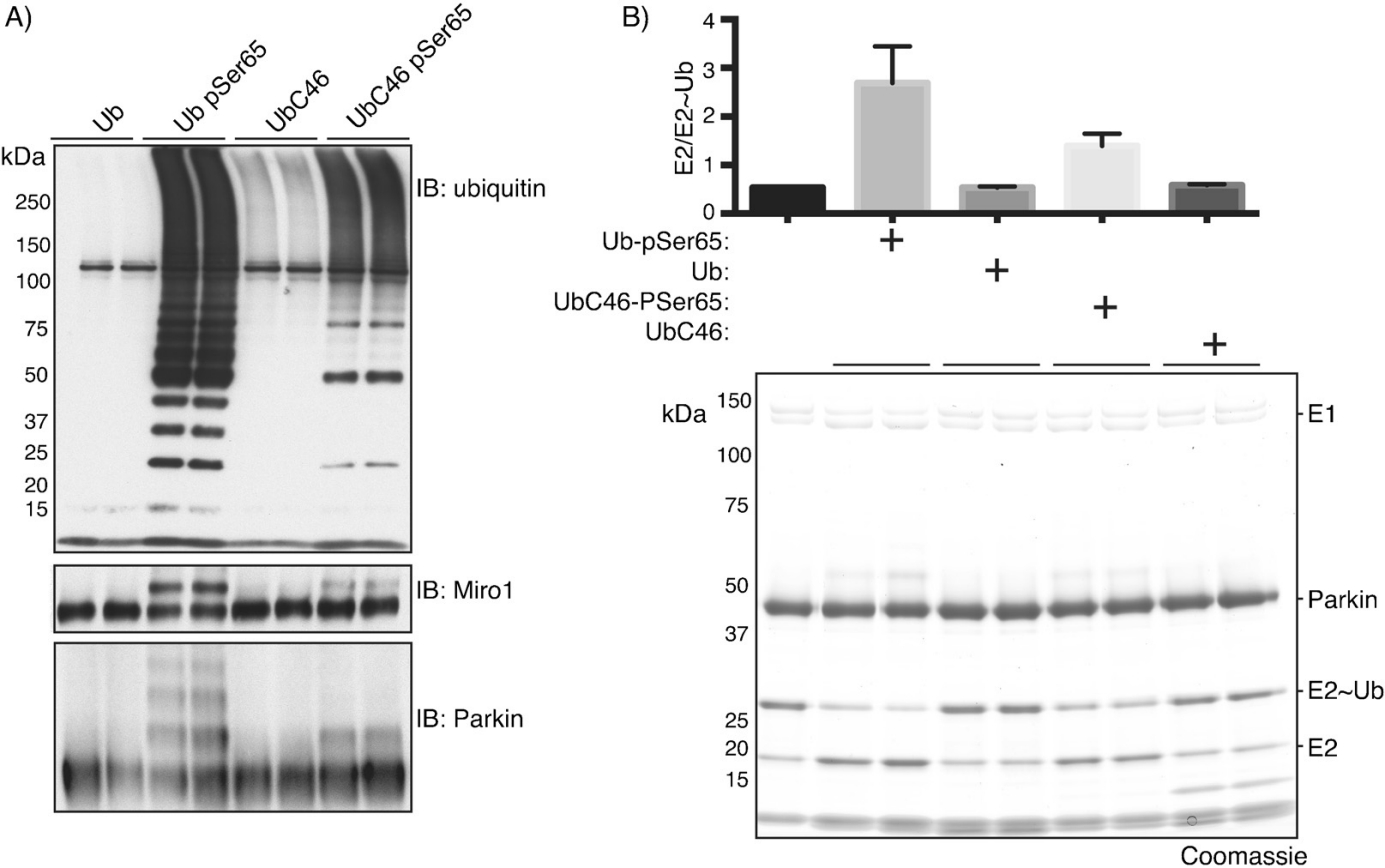
Biochemical analysis of activation of Parkin E3 ligase by phospho-Ser65-ubiquitin. A) Top: Ub (anti-Flag) immunoblot of Parkin-mediated polyubiquitin chain formation in the presence of the indicated Ub species. Ub: wild-type ubiquitin; Ub-pSer65: wild-type ubiquitin enzymatically phosphorylated by recombinant PINK1;^10^ UbC46 and UbC46-pSer65: synthetic non-phosphorylated and phosphorylated ubiquitin generated by ligation of biosynthetic and synthetic precursors. Middle: Miro1 (anti-SUMO) immunoblot demonstrates Parkin-mediated ubiquitylation of His-SUMO-Miro1 in the presence of the various Ub species. Bottom: anti-Parkin immunoblot demonstrates the extent of autoubiquitylation in the presence of the indicated Ub species. (B) Top: quantification of Parkin-dependent E2 discharge mediated by the indicated Ub species. The activity was assessed by change in UbcH7/UbcH7-Ub ratio. Bottom: the E2-charging reaction was performed in the presence of Ube1, UbcH7 and FLAG-ubiquitin with magnesium acetate and ATP followed by addition of indicated Ub species. Reaction mixtures were subjected to SDS-PAGE and Coomassie staining.

To further validate the integrity of our phosphorylated ubiquitin we proceeded to solve its structure. First we screened conditions for the crystallisation of UbC46-pSer65. After 5 weeks at 18 °C, crystals were obtained in drops equilibrated against reservoir buffer composed of 50 mM sodium cacodylate (pH 5.0) and 25 % PEG 4000. Diffraction data were collected at Diamond Light Source, Oxfordshire, UK. Phases were obtained by molecular replacement with a previously solved ubiquitin structure (PDB ID: 1UBQ)^20^ as a search model. Structure refinement was carried out with Phoenix, and model building was carried out with COOT. The final structure was refined to 1.54 Å (*R*_work_ 0.139, *R*_free_ 0.173; Table S1) and submitted the Protein Data Bank (PDB ID: 4ZPZ).

In our structure there are two symmetry-related Ub molecules in the asymmetric unit (RMSD 0.081 Å) in the *P*3_1_ space group (Figure 3 A); this differs from the previously solved Ub-pSer65 structure.^10^ The observed symmetry permitted assignment of the *P*3_1_21 space group. However, the two Ub molecules form a covalently linked oxidised homodimer (apparent from the contiguous electron density), linked by the two cysteines arising from the Ala46Cys mutation (Figure 3 D). Consequently we assigned the *P*3_1_ space group. Wild-type non-phosphorylated ubiquitin has previously been crystallised in the *P*3_1_21 space group (PDB ID: 4HK2) but our structure is based on a different crystal form. Structural conservation between molecule 2 in our structure and the reported structure of phospho-ubiquitin was high (main chain RMSD 0.659 Å). The β-strand slippage that gives rise to a reported minor conformation species^10^ was not observed, thus indicating that our structure also represents the major conformation, despite having a different crystal form. The electron density for pSer65 was clearly visible, thus allowing unambiguous assignment of the pSer65 rotomer (Figure 3 B). Although the orientation of pSer65 largely mirrors that in the previous structure, the hydrogen bond between a phosphate oxygen and the backbone amide nitrogen of Gln62 was less significant (3.8 vs. 3.3 Å). In our structure, which is at a higher resolution, the γ-oxygen forms a more favourable H-bond with the amide nitrogen of Gln62 thereby forming an H-bond analogous to that in the wild-type ubiquitin structure^10^ (Figure 3 C). The orientation of the phosphate thus appears to be dictated by a solvent-mediated hydrogen-bond network involving Thr66. A water molecule bridges a phosphate oxygen of pSer65 and the main-chain amide nitrogen of Thr66. This water molecule also hydrogen bonds with a second water that forms a hydrogen bond with the hydroxyl group of Thr66. All the described hydrogen bonds have favourable geometries (2.5–3.2 Å; Figure 3 C). However, pSer65 does exist at a packing interface and is directed towards His68 of an adjacent Ub molecule. We cannot therefore exclude the possibility that the orientation is an artefact of crystallisation, or that this interaction is maintained in solution.

**Figure 3:**
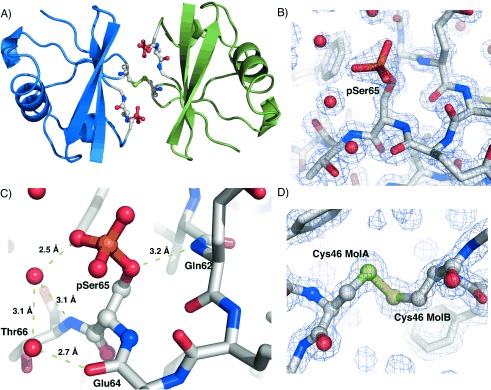
X-ray crystal structure of UbC46-pSer65. A) Two disulfide-linked UbC46-pSer65 molecules in the asymmetric unit; pSer65 and Cys46 are depicted in ball and stick representation. B) Stick representation pSer65 residue; water molecules are depicted as red spheres. The 2 *F*_o_−*F*_c_ electron density map (1.5 *σ*) is shown as a blue mesh. C) Hydrogen bonding network around pSer65. D) Disulfide bond between Cys46 of two Ub molecules in the asymmetric unit. Contiguous electron density is clearly evident between the γ-sulfur atoms of Cys46 (2 *F*_o_−*F*_c_ electron density map at 1.5 *σ*).

In summary, we report a strategy that provides a platform for the production of ubiquitin that can be synthetically modified within the C-terminal 30 residues. The N-terminal 1–45 residues are prepared biosynthetically and are amenable to conventional site-directed mutagenic strategies. Furthermore, unnatural functionality could be incorporated into this region by stop-codon-suppression techniques.^21^ Such techniques allow the genetic incorporation of numerous post-translational modifications, including acetylation and phosphorylation.^13^, 16a Ubiquitylation can also be genetically directed, thus enabling the generation of mixed-linkage chains of a desired topology.16b, 17b, 22 The strategy reported here should therefore complement these approaches to allow the production of complex polyubiquitin molecules containing molecule- and site-specific phosphorylation(s). Importantly, such preparations could be carried out entirely without ubiquitin-modifying enzymes or kinases, which are often non-specific, inefficient or simply unknown. The strategy should facilitate the study of the combined effects of phosphorylation and ubiquitylation and thus provides a powerful approach to addressing outstanding questions on the specific interplay of Ub-pSer65 and polyubiquitin chain topology in the context of activation of the PINK1–Parkin pathway, as well as its cellular significance.

## Supporting Information

As a service to our authors and readers, this journal provides supporting information supplied by the authors. Such materials are peer reviewed and may be re-organized for online delivery, but are not copy-edited or typeset. Technical support issues arising from supporting information (other than missing files) should be addressed to the authors.

miscellaneous_information
