# Supplementary material for: A Versatile Strategy for the Semisynthetic Production of Ser65 Phosphorylated Ubiquitin and Its Biochemical and Structural Characterisation
Source: Chembiochem. 2015 Jun 18;16(11):1574–9. doi: 10.1002/cbic.201500185 (PMC4581463; doi:10.1002/cbic.201500185)
Supplement: Supplementary file 1 — miscellaneous_information [file cbic0016-1574-sd1.pdf]

CHEMBIOCHEM

Supporting Information

A Versatile Strategy for the Semisynthetic Production of Ser65 Phosphorylated Ubiquitin and Its Biochemical and Structural Characterisation

Cong Han, Kuan-Chuan Pao, Agne Kazlauskaite, Miratul M. K. Muqit, and Satpal Virdee*[a]

cbic_201500185_sm_miscellaneous_information.pdf

General Materials

All DNA constructs were verified by DNA sequencing, which was performed by The Sequencing Service, School of Life Sciences, University of Dundee, using DYEnamic ET terminator chemistry (Amersham Biosciences) on Applied Biosystems automated DNA sequencers. DNA for bacterial protein expression was transformed into *E. coli* BL21 DE3 RIL (codon plus) cells (Stratagene). All cDNA plasmids, antibodies and recombinant proteins employed in this study are available to request through our reagents website (https://mrcppureagents.dundee.ac.uk/).

General Methods

LC-MS was carried out with an Agilent 1200 LC-MS system fitted with a Max-Light Cartridge flow cell coupled to a 6130 Quadrupole spectrometer. The solvent system consisted of 0.05 % trifluoroacetic acid (TFA) in H_2O as buffer A, and 0.04 % TFA acid in acetonitrile (MeCN) as buffer B. Protein UV absorbance was monitored at 214 and 280 nm. An Agilent ZORBAX 300SB-C3 5um, 2.1 x 150mm column was employed for proteins unless otherwise stated. Protein MS acquisition was carried out in positive ion mode and total protein masses were calculated by deconvolution within the MS Chemstation software (Agilent Technologies). Semi-preparative peptide HPLC was carried out on a Dionex Ultimate system with Thermo Biobasic C4 21.2 x 250 mm column at a flow rate of 10 ml min^{-1}. All solvents and reagents were purchased from Sigma Aldrich or VWR unless otherwise stated.

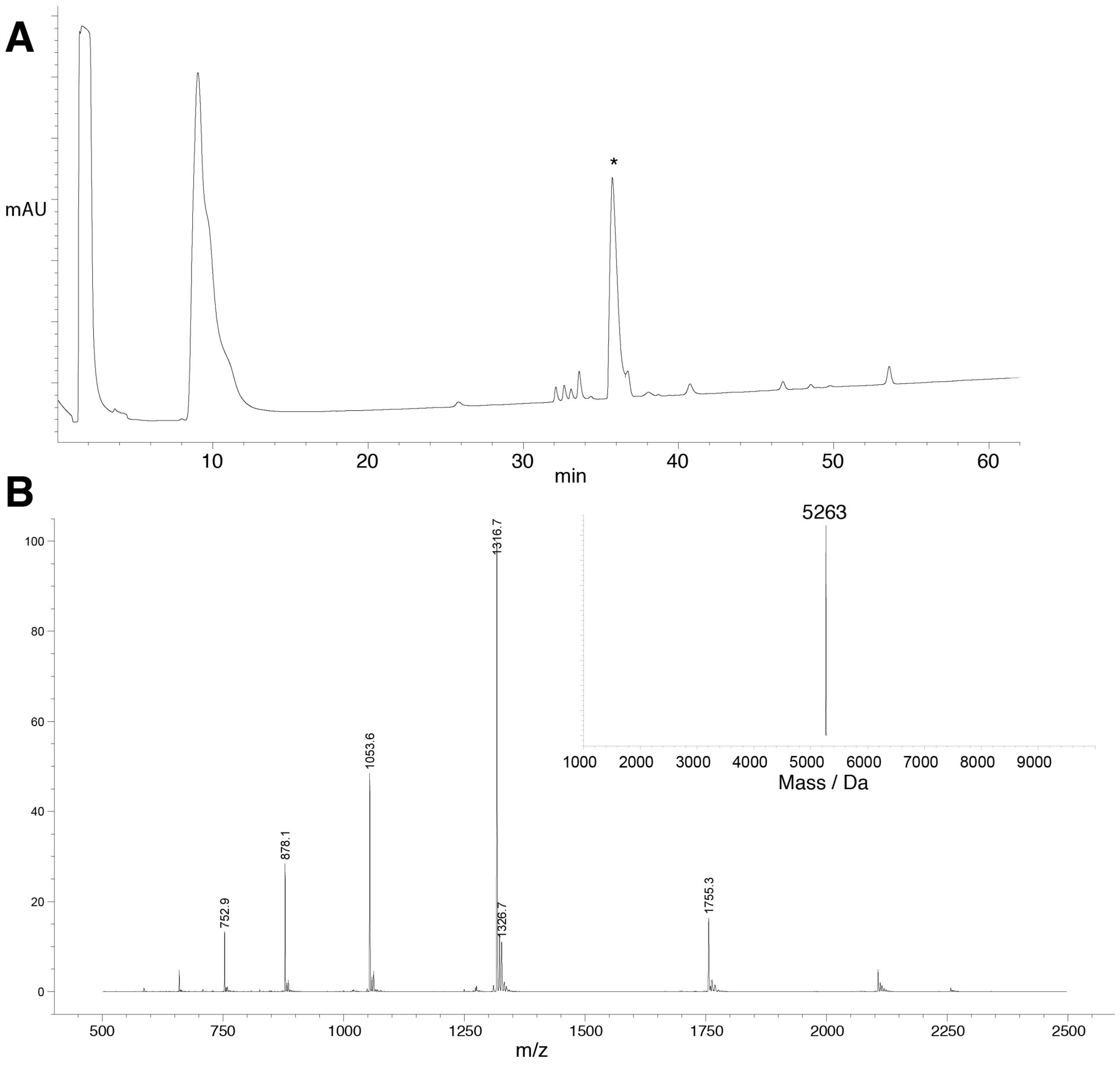

Figure S1. LC-MS characterization of Ub1-45-SR in ligation buffer. (A) HPLC chromatogram of Ub1-45-SR. Peak corresponds to the MPAA thioester formed from *in situ* transthioesterification with MPAA (B) ESI-MS spectra for Ub1-45-SR. Inset, deconvoluted spectrum. Expected mass 5264.15 Da; found 5263 Da.

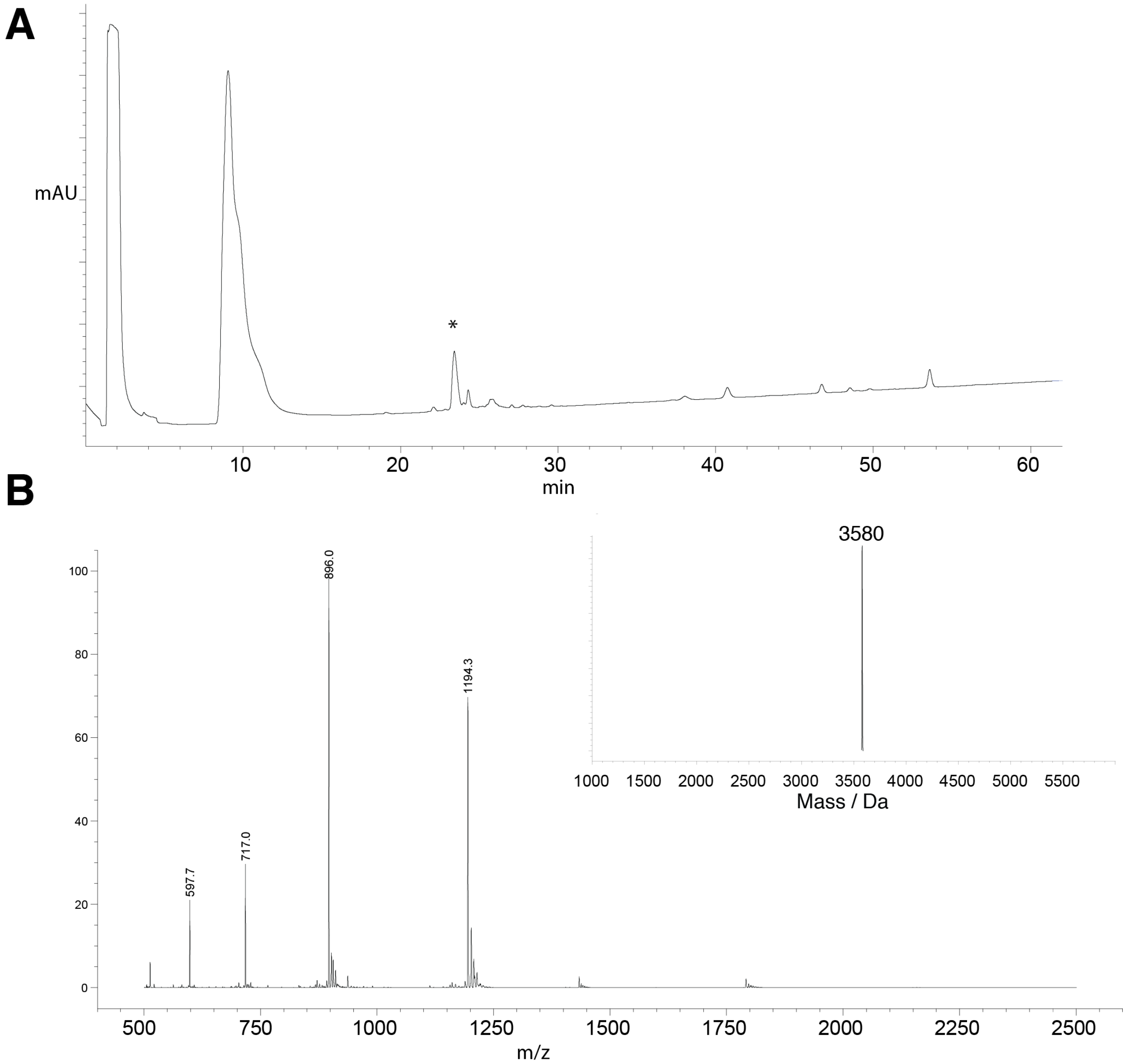

Figure S2. LC-MS characterization of UbC46-76-pSer65 in ligation buffer. (A) HPLC chromatogram of UbC46-76-pSer65. (B) ESI-MS spectra for UbC46-76-pSer65. Inset, deconvoluted spectrum. Expected mass 3580.98 Da; found 3580 Da.

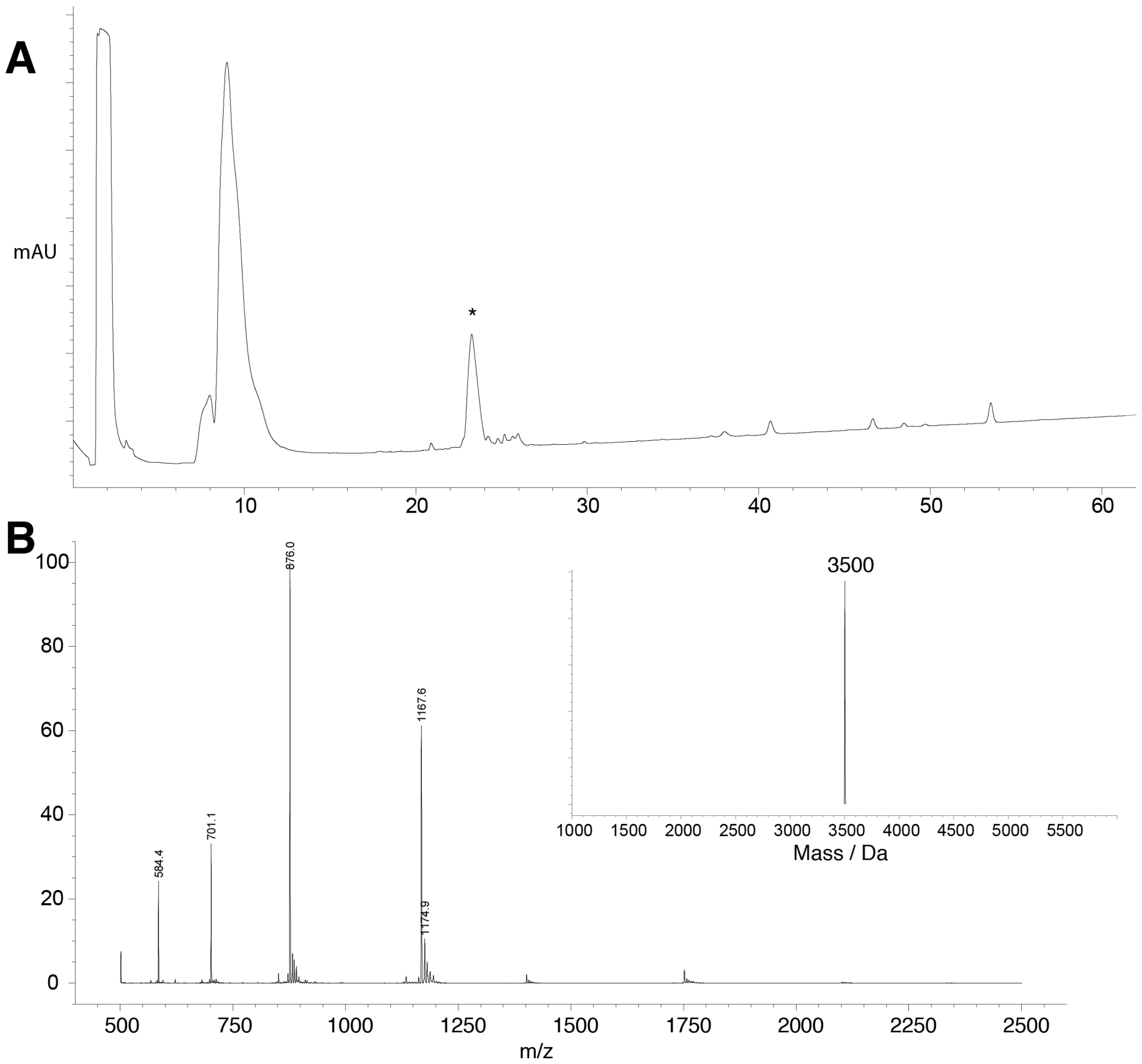

Figure S3. LC-MS characterization of UbC46-76 in ligation buffer. (A) HPLC chromatogram of UbC46-76. (B) ESI-MS spectra for UbC46-76. Inset, deconvoluted spectrum. Expected mass 3500.98 Da; found 3500 Da.

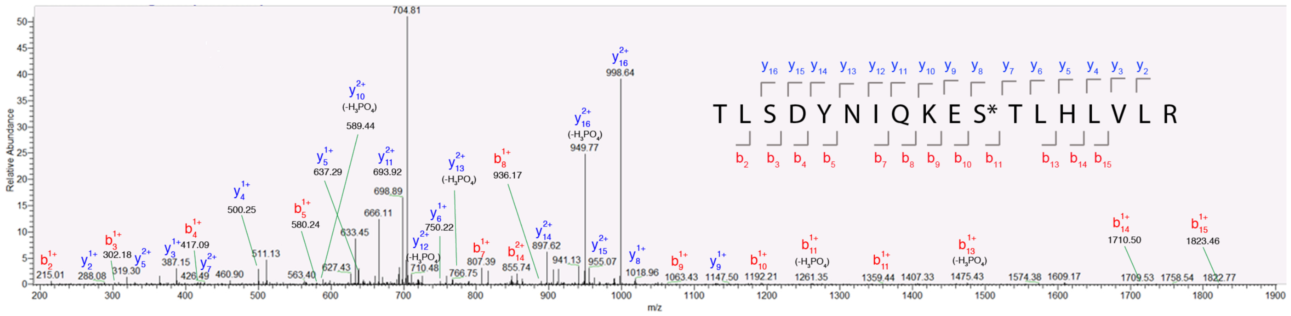

Figure S4. Annotated MS2 spectrum for the single phosphopeptide identified. S* corresponds to phosphoserine at position 65 which was detected intact, and as neutral loss (-H_3PO_4). Missed cleavage was observed at Lys63.

Preparation of Ub-1-45-SR

The plasmid *pTXB1-Ub-1-45* was prepared by PCR deletion of the bases coding for resides 46-76 in the plasmid *pTXB1-Ub-1-76*[1] by PCR. Deletion was confirmed by DNA sequencing. *pTXB1-Ub-1-45* was transformed into ER2566 cells (New England Biolabs) and Ub-1-45-SR was expressed and purified as previously described for full-length ubiquitin[1].

Peptides UbC46-76 and UbC46-76-pSer45

N-terminal cysteine peptides were sourced from Pepceuticals Ltd, UK on a 5 mg scale for UbC46-76 and a 25 mg scale for UbC46-76-pSer65. Requested purity level was >95%.

Expressed protein ligation

Ub-1-45-SR (8.0 mg, 1.53 μmol) was ligated to UbC46-76-pSer65 (4.8 mg, 1.35 μmol) and UbC46-76 (4.7 mg, 1.35 μmol) in 1.19 ml of ligation buffer (200 mM Na_2HPO_4 buffer at pH 6.8 containing 6 M guanidinium chloride [GdmCl], 100 mM mercaptophenylacetic acid [MPAA] and 60 mM *tris* [2-carboxyethyl] phosphine [TCEP]). The reaction was monitored by analytical RP-HPLC (10–50 % buffer B; buffer B = 0.1% TFA in MeCN, over 60 min, 0.3 ml/min). The reaction was ~90% complete after 2 hr. The full-length ubiquitin products were

purified by semi-preparative RP-HPLC (10–50 % buffer B; buffer B = 0.1% TFA in MeCN, over 120 min, 10 ml/min). Fractions containing the protein were verified by LC-MS and lyophilized (UbC46-pSer65 yield: 5.2 mg, 0.60 μmol). Peptides were folded by dissolution in denaturing buffer (200 mM Na_2HPO_4 pH 7.5, 6 M GdmCl) and overnight dialysis against phosphate buffered saline containing 0.5 mM TCEP.

Desulfurization of UbC46-pSer65 to generate native Ub-pSer65

lyophilized UbC46-pSer65 (1 mg) was dissolved in 1 ml of denaturing buffer (200 mM Na_2HPO_4 pH 6.9, 6 M GdmCl, 0.5 mM TCEP). Next, 335 μL TCEP solution (1 M in 4 N NaOH), 13 μL aqueous glutathione solution (500 mM) and 6.5 μL aqueous VA-044 (2,2'-azobis[2-(2imidazolin-2-yl)propane]dihydrochloride; 200 mM; Wako chemicals) were added. All solutions were prepared fresh and purged with argon. The reaction was then incubated for 3 h at 37 °C, 600 rpm in an Eppendorf Thermomixer. LC-MS analysis confirmed the presence of a single product with a mass -32 Da corresponding to conversion of C46 to the native A46 residue thereby generating native Ub-pSer65. Removal of small molecule reaction components and folding was achieved by dialysis against folding buffer (10 mM Tris pH 7.5, 100 mM NaCl). Sample was subsequently passed through a desalting column.

In-gel trypic digestion and phospho mapping by LC-MS/MS

UbC46-pSer65 was reduced with DTT (5 mM) and boiled in 1X LDS sample buffer (Life Technologies). Sample was then alkylated with iodoacetamide (20mM) and incubated at room temperature in the dark for 30 min. DTT (20mM) was then added and the sample was resolved by SDS-PAGE and stained with Instant Blue (Expedeon). The gel band was then cut into ~1mm cubes. In brief, the gel pieces were washed with water and dehydrated with

acetonitrile (MeCN). Sample was then digested with trypsin in 50 mM Triethylammonium bicarbonate. Sample was then dehydrated and resuspended in 0.1 % aqueous TFA and extracted with MeCN. Supernatant was then evaporated under reduced pressure and LC-MS/MS analysis was performed on an LTQ Velos Pro instrument (Thermo Scientific) coupled to an Ultimate nanoflow HPLC system (Dionex). A gradient running from 3 % solvent A to 90 % solvent B over 21 min was applied (solvent A = 0.1 % formic acid in H_2O; solvent B = 0.08% formic acid in 80% MeCN). Fragment ions were generated by CID. Spectra obtained were then searched against an in-house database with the inclusion of phosphate (+80 Da) and neutral loss of phosphate (-98 Da) as variable modifications. A single phosphopeptide was unambiguously identified corresponding to TLSDYNIQKES*TLHLVLR, (i.e. phosphoserine at position 65 and missed cleavage at Lys63). The scan number of a corresponding MS2 spectrum was identified and which was then visualized in Xcalibur (Thermo Scientific). Fragment masses were manually annotated as depicted in Figure S4.

Parkin ubiquitylation assay

Wild-type Parkin (2 μg) was added to ubiquitylation assay component mastermix [50mM Tris/HCl (pH 7.5), 5 mM MgCl2, 0.12 μM ubiquitin E1, 1 μM UbcH7, 2 μg of His6–SUMO-Miro1 and 2 mM ATP]. Each reaction contained 0.05mM ubiquitin comprising 25 μg of FLAG-ubiquitin (Boston Biochem) mixed with 5 μg of Ub, Ub-pSer65, UbC46 or UbC46-pSer65. Ubiquitylation reactions were carried out as previously described[2]. Ubiquitylation reactions were subjected to analysis by immunoblotting as follows: ubiquitin (anti-FLAG antibody), Parkin (anti-Parkin antibody) and Miro-1 (anti-SUMO1 antibody).

Parkin-mediated E2~Ub discharge assay

The E2-charging reaction was undertaken as previously described and comprised Ube1, UbcH7, 50 mM Hepes (pH 7.5) and 10 μM FLAG-ubiquitin in the presence of 2 mM magnesium acetate and 0.2 mM ATP[2]. After an initial incubation of 60 min at 30°C, wild-type Parkin was added in the presence of Ub, Ub-pSer65, UbC46 or UbC46-pSer65. The reaction was allowed to continue for a further 10 min at 30°C. Reactions were terminated and were subjected to SDS/PAGE in the absence of any reducing agent. Gels were stained using InstantBlue and band quantification was carried out using a Licor imaging system (700 nm channel).

Crystallization of UbC46-pSer65

UbC46-pSer65 was buffer exchanged into 10 mM Tris-HCl pH 7.5, 0.5 mM TCEP using a desalting column. UbC46-pSer65 crystals were grown in hanging drops containing 1.5 μl each of protein (2 mg ml^{-1}) and a reservoir solution of 50 mM sodium cacodylate pH 5.0, 25% PEG 4000 at 18 °C. Crystals appeared after 4 weeks and grew to their final size within 5 weeks. Crystals were harvested in a cryoprotectant solution (50 mM sodium cacodylate pH 5.0, 25% PEG 4000, and 15% PEG 400) before flash-freezing in liquid nitrogen. X-ray diffraction data were collected on beamline I03 at the Diamond Light Source, UK. Data were processed with iMOSFLM[3]. The structure was solved by molecular replacement using PHASER[4] with native ubiquitin (PDB 1UBQ, truncated at residue R72) as a search model[5]. The model was adjusted manually using COOT[6], and refined using Phenix[7] and PDB_REDO[8]. Apparent electron density was visible for residues 1-72 for molecule 1 and 1-73 for molecule 2. For data collection and refinement statistics see Table S1.

	UbC46-pSer65
**Data Collection**	
Space group	$P3_1$
Cell dimensions	
a, b, c (Å)	41.7, 41.7, 69.83
α, β, γ (°)	90, 90, 120
Wavelength (Å)	0.9797
Resolution (Å)	25.1-1.54 (1.62-1.54)
R_{merge}	0.067 (0.333)
$I / \sigma I$	10.2 (3.2)
Completeness (%)	99.7 (98.8)
Redundancy	3.1 (2.8)
**Refinement**	
Resolution (Å)	25.1-1.54
No. reflections	19062
R_{work} / R_{free}	0.139/0.173
No. atoms	
Protein	1199
Water	145
B-factors	
Protein	15.9
Water	19.8
R.m.s. deviations	
Bond length (Å)	0.0145
Bond angles (°)	1.825
Ramachandran plot (%)	
Most favored	98.3
Allowed	1.7
Disallowed	0.0

Values in parentheses are for the highest resolution shell.

Table 1. Data collection and refinement statistics.

Supplementary references

[1] S. Virdee, Y. Ye, D. P. Nguyen, D. Komander, J. W. Chin, *Nature Chemical Biology* **2010**, *6*, 750-757.

[2] A. Kazlauskaite, C. Kondapalli, R. Gourlay, D. G. Campbell, M. S. Ritorto, K. Hofmann, D. R. Alessi, A. Knebel, M. Trost, M. M. K. Muqit, *The Biochemical journal* **2014**, *460*, 127-139.

[3] T. G. Battye, L. Kontogiannis, O. Johnson, H. R. Powell, A. G. Leslie, *Acta Crystallogr D Biol Crystallogr* **2011**, *67*, 271-281.

[4] A. J. McCoy, R. W. Grosse-Kunstleve, P. D. Adams, M. D. Winn, L. C. Storoni, R. J. Read, *J Appl Crystallogr* **2007**, *40*, 658-674.

[5] S. Vijay-Kumar, C. E. Bugg, W. J. Cook, *Journal of molecular biology* **1987**, *194*, 531-544.

[6] P. Emsley, B. Lohkamp, W. G. Scott, K. Cowtan, *Acta crystallographica. Section D, Biological crystallography* **2010**, *66*, 486-501.

[7] P. D. Adams, P. V. Afonine, G. Bunkoczi, V. B. Chen, I. W. Davis, N. Echols, J. J. Headd, L. W. Hung, G. J. Kapral, R. W. Grosse-Kunstleve, A. J. McCoy, N. W. Moriarty, R. Oeffner, R. J. Read, D. C. Richardson, J. S. Richardson, T. C. Terwilliger, P. H. Zwart, *Acta crystallographica. Section D, Biological crystallography* **2010**, *66*, 213-221.

[8] R. P. Joosten, F. Long, G. N. Murshudov, A. Perrakis, *IUCrJ* **2014**, *1*, 213-220.
